# Gender and antimicrobial resistance: a conceptual framework for researchers working in livestock systems

**DOI:** 10.3389/fvets.2024.1456605

**Published:** 2025-01-28

**Authors:** Fiona Emdin, Alessandra Galiè, Arshnee Moodley, Susan Rogers Van Katwyk

**Affiliations:** ^1^Antimicrobial Resistance Policy Accelerator, Global Strategy Lab, York University, Toronto, ON, Canada; ^2^International Livestock Research Institute, Nairobi, Kenya; ^3^Department of Veterinary and Animal Sciences, University of Copenhagen, Frederiksberg, Denmark

**Keywords:** gender, antimicrobial resistance, livestock, framework, perspective

## Abstract

**Background:**

To promote gender mainstreaming in future AMR research projects and policy implementation within livestock and other systems, researchers need to embrace gender-responsive research methodologies. Ignoring gender considerations can lead to unsustainable interventions and exacerbate existing equity gaps. Incorporating gender analysis is crucial for identifying data collection needs and opportunities to develop gender-responsive research programs and policies.

**Objectives:**

We have developed a conceptual framework and a set of research questions designed to enhance the gender-responsiveness of AMR research in livestock systems.

**Methods:**

A narrative review previously identified three key entry points for gender dynamic impacting AMR in agricultural systems: gendered antimicrobial resistance exposure, gendered antimicrobial use and gendered outcomes of antimicrobial resistance infections. This information was then analyzed using a health system gender framework. Combining these insights, we developed a comprehensive list of research questions.

**Results:**

We developed comprehensive list of gender-related questions. Given the limited understanding of how gender dynamics and norms influence AMR, we have primarily proposed qualitative, exploratory questions. These questions are categorized into two types: integrated and strategic. Integrated questions offer a deeper understanding of gender dynamics and norms in livestock systems with the aim of improving them, while strategic questions focus on gender-related issues in livestock as entry points, highlighting some of the mechanisms behind these gender issues to progress towards gender equality.

**Conclusion:**

As gender-analysis in livestock research gains prominence, there is an increasing expectation for AMR researchers to integrate gender considerations into their work. This framework provides a starting point for researchers aiming to enhance gender inclusivity and considerations in AMR research within livestock systems. The next phase of our project will involve applying this framework in the field, where a real-life application will enable its validation and further refinement.

## 1 Introduction: the link between gender and AMR research in livestock systems

Antimicrobial resistance (AMR) is a growing public health threat, which like most public health issues, is inequitably distributed across countries, populations, and economic regions (1–4), and is influenced by social, economic, and cultural factors, including gender dynamics and norms (5). A recent study estimated that in 2019, bacterial AMR contributed to almost 5 million deaths (6) and in 2020, the World Health Organization (WHO) estimated that AMR has caused at least one-third as many deaths as COVID-19 (7).

**Figure 1:**
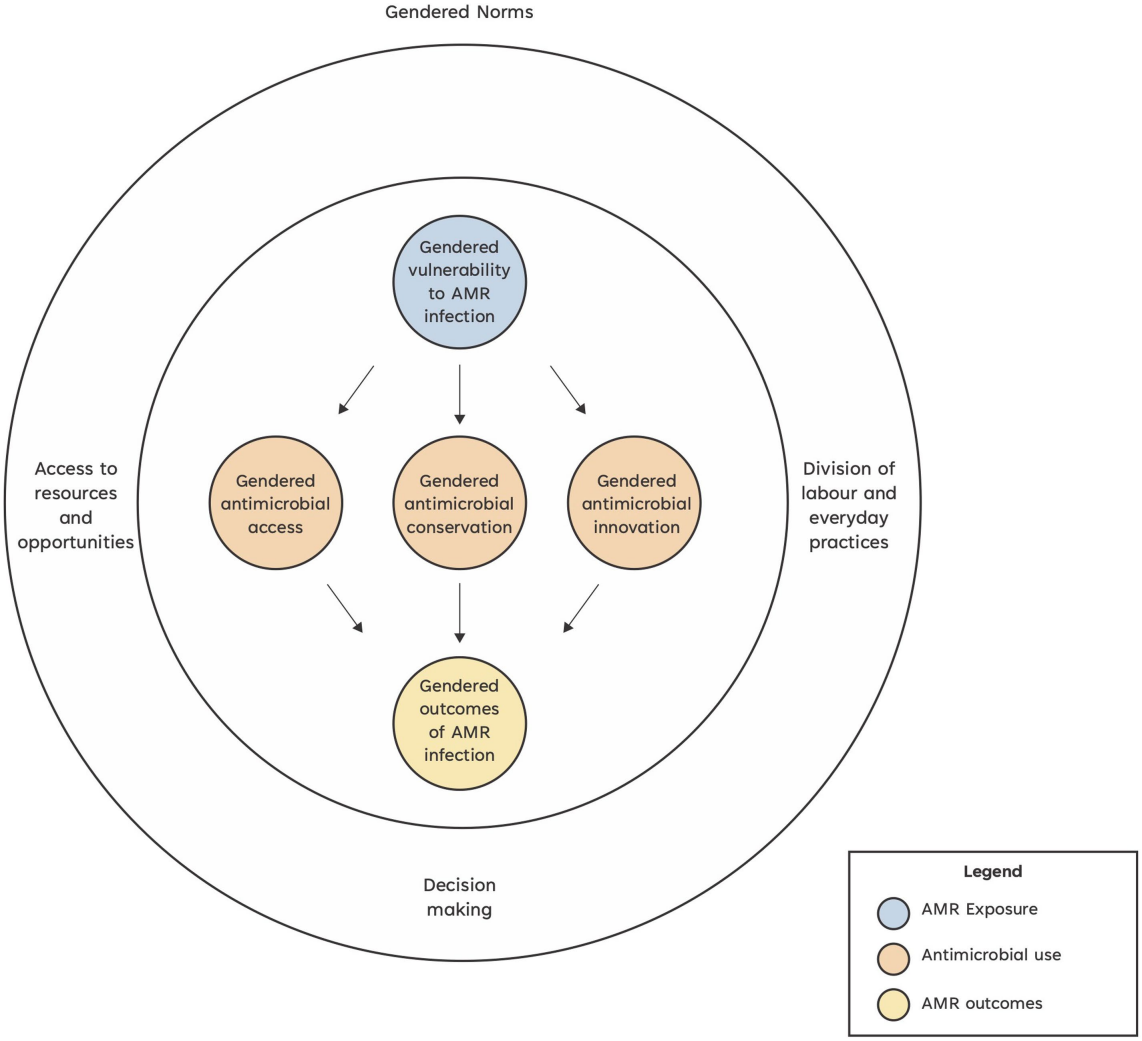
Conceptual framework of how gender issues might impact antimicrobial resistance in livestock systems.

In livestock systems, gender norms are known to result in inequitable impacts (8). We define gender norms as the spoken and unspoken rules that establish what is appropriate for a woman or a man (based also on their other individual characteristics such as age, ethnicity, and religion) to do, believe, say, access, own, benefit, and claim in various livestock systems and contexts (9). Gender norms shape gender dynamics, which in turn influence the intra-household division of labor, access to assets and decision-making, among other impacts. These arrangements may influence how different household members are exposed to pathogens. Gender dynamics shape: (1) who does what in the household livestock management affecting, for example, the extent to which people are exposed to resistant pathogens; (2) who has decision-making power in livestock-related activities influencing, for example, who decides to utilize antimicrobials or alternative remedies, with women often less able to purchase antibiotics and vaccines for animals they own or manage (10); (3) who can use and access resources [affecting for example, who has money to purchase antimicrobials or who accesses information about available alternatives and support services such as veterinarians (9, 11, 12)]; and (4) who can take advantage of opportunities (for example, attend a training session on AMR) or access new technologies (e.g., genetically modified stock) which might reduce antibiotic use. These differences in roles and opportunities ultimately shape the ways in which resistant infections impact the lives of women and men (13) (Figure 1).

In addition to shaping who is impacted by AMR, and how they experience these impacts, gender dynamics and norms also impact the effectiveness of interventions to reduce AMR. To reach the appropriate household member these interventions need to incorporate a gender lens and be tailored to the activities and roles they undertake, and to the needs and opportunities they may have regarding access to animal health services, medicines, and information. Not only will gender-responsive AMR research reduce AMR impacts, but it will also make interventions more effective, ultimately benefiting whole communities. Economic analyses have shown that empowering women producers can bring economic benefits to households and communities; with increased control over agricultural income resulting in higher spending on food and education (14).

To build gender-responsive AMR research programs and policies that address underlying power structures and norms that may disadvantage some social groups (often women and girls)—and progress towards equitable AMR interventions, more evidence is needed to identify the research gaps related to gender issues and AMR in livestock systems (15). Available data is rarely disaggregated by gender (16) thereby overlooking quantitative assessments of existing gender-based disadvantage and of gendered needs and opportunities. There has also been a limited focus on gender dynamics that lead to disadvantages related to AMR, particularly outside the human health context; few National Action Plans on AMR include gender-related provisions or consider gender as a driver of inequity in AMR-related outcomes. Recently, gender has been identified as a research priority in the One Health Priority Research Agenda on AMR which calls for better consideration of gender as a priority, cross-cutting challenge with the potential to impact all other research priorities (17).

To address this evidence gap around AMR and gender dynamics in livestock systems, we have created a conceptual framework and list of research questions which can be used to improve the gender-responsiveness of AMR research in livestock systems. We previously completed a narrative review which identified three key entry points for gender dynamic impacts on AMR in agricultural systems: gendered antimicrobial resistance exposure, gendered antimicrobial use, and gendered outcomes of antimicrobial resistance infections (18). We then applied Morgan et al.’s health system analysis gender framework (8) to the information derived from this narrative review to develop a novel conceptual framework exploring the role of gender and the impact of gender dynamics on AMR in livestock systems. Morgan et al. argue that gender as a driver of inequity in health systems can be understood by how power is constituted and negotiated (8). They assert that gender power relations can be understood by asking who has what (access to resources and access to opportunities); who does what (the division of labor and everyday practices); who decides (decision-making) and how values are defined (social and gender norms, ideologies, beliefs, and perceptions). Since gender and social norms ultimately affect access to resources and opportunities, division of labor and everyday practices, we incorporated this gender lens as an overarching element of our Conceptual framework of how gender issues might impact AMR in livestock systems (Figure 1). We define gender analysis as an analysis of the gender identities and power dynamics that affect the ways in which women and men are positioned in society and the distribution of resources, opportunities, constraints, and power in each context. Such analysis needs to consider how other individual characteristics (e.g., age, ethnicity, caste, religion, marital status etc.) affect the commonalities and differences between and among women and men. It also needs to appreciate the systemic and relational, as well as the individual dimensions at play.

We defined AMR exposure as exposure to antimicrobial resistant pathogens during livestock production; antimicrobial use as access to antimicrobials, use of antimicrobial alternatives such as vaccines and adoption of innovations like novel antimicrobials or technologies; and AMR outcomes as the consequences of AMR infections within livestock systems including productivity, economic, health, and nutritional impacts.

For each of the three key gender entry points in livestock systems in our conceptual framework (AMR exposure, antimicrobial use (AMU) and AMR outcomes) we developed a list of integrated and strategic research questions. Integrated questions are those with a bioscience entry-point (19). They allow a deep understanding of gender dynamics and norms in livestock systems with the goal of improving the gender-responsiveness of bioscience questions and interventions. An example of an integrated question would be “*who is authorized to use antimicrobials in livestock production?*” On the other hand, strategic questions are those that have a gender-related question as entry point with the goal of progressing towards gender equity in livestock systems. An example of a strategic question would be “*which livestock producers are willing but unable to use antibiotics, and what are the barriers that need to be overcome*?” Strategic questions are key to identify gendered aspirations which in turn are necessary to ensure interventions do not just reproduce current (inequitable) gendered roles - thereby reproducing gender-based disadvantage - but rather, leverage interventions to move towards more gender equitable arrangements. Our research questions are the first step to conceptualizing how gender dynamics and norms impact livestock systems and to identify potential entry points to address gender issues and AMR related outcomes in these systems.

## 2 Developing a research framework to improve the gender-responsiveness of AMR research in livestock systems

The influence of gender on AMR exposure (20, 21), patterns of antimicrobial use (22, 23), and outcomes from AMR infections (24) is well-documented in human health. These three aspects of AMR – exposure, antimicrobial use, and outcomes—can also be gendered in the context of livestock systems. These dimensions are also interlinked: not only do gender inequities drive gendered AMR exposure, antimicrobial use, and AMR policy outcomes, but gendered AMR exposures can also result in gendered antimicrobial use which in turn may drive gendered AMR policy outcomes. As such, these three aspects should be key considerations in any research conducted in this domain.

### 2.1 Antimicrobial resistance (AMR) exposure

Gendered AMR exposure refers to inequitable and unequal risk faced by people of different genders to antimicrobial resistant pathogens in livestock production, which arise directly through contact with animals and their waste, or indirectly, through activities required for production such as disinfection of farm equipment or the farm environment, preparing forage and feeds. Gender norms that influence labor division and daily practices can influence such exposure to resistant pathogens. For example, in Cambodia, women and children, typically responsible for managing animal manure and slaughter products, exhibit higher rates of AMR enteric pathogens (25).

### 2.2 Antimicrobial use (AMU)

In livestock systems, antimicrobial use is influenced by several factors including access to antimicrobials (which we have called “*access*”), use of antimicrobial alternatives such as vaccines (which we refer to as “*conservation*”), and adoption of innovations like novel antimicrobials or technologies that decrease the need for antimicrobials or reduce disease incidence (which we have termed “*innovation*”) (26). Access to antimicrobials, alternatives, and innovations (AAIs) is greatly shaped by gender dynamics and norms, which vary across different local contexts. It is dependent, for example, on a person’s ability to access information on available AAIs, and to then obtain AAIs from animal health service providers or drug sellers. Gender norms and dynamics strongly affect who accesses information and public spaces (such as agrovets). Gender norms can obstruct access to agricultural extension officers, who provide advice on disease treatment and antimicrobial use. For example, women farmers in Ghana were less likely to seek advisory services since most extension officers are men and socio-cultural traditions often prevent them from meeting with them on their own (27, 28). AAIs need to be affordable, also, to be obtained. Affordability is influenced by gender dynamics and norms around who in the household controls income (10). For instance, women farmers have reported having limited authority over financial decisions regarding the animals they manage (29) which may restrict their ability to purchase antimicrobials. A person’s ability to utilize the obtained AAIs to treat animal diseases is influenced, in turn, by gender dynamics around, for example, who takes decisions on livestock and who oversees treating the livestock.

Antimicrobial conservation may be impacted, also by gendered differences in education or training on prophylactic health products or antimicrobial alternatives like vaccines or prebiotics in animal production (30, 31). For example, among pig and chicken farmers in Cambodia, men were more likely to be aware of AMR and demonstrated more appropriate use of antimicrobials (32). Gendered use of novel antimicrobials or antimicrobial reducing innovations and technologies (e.g., improved genetic stock) is also well documented (33, 34).

### 2.3 AMR outcomes

The consequences of AMR infections within livestock systems may be experienced differently based on gender; these AMR outcomes can affect productivity, economic stability, health, and nutrition in different ways. Studies indicate that in Sub-Saharan Africa, diseases increase the caregiving responsibilities of women, leading to greater household vulnerability to food insecurity. For example, a study in Tanzania showed that a woman spent 45% less time on farming activities when their husbands were ill (35). Additionally, it has been reported that in cases where households experienced the death of a male head, women lost their livestock assets to other relatives in 33% of the instances, further highlighting the gendered impacts of AMR on socio-economic outcomes (36). Health emergencies and disease outbreaks, which may be further exacerbated by AMR, can affect genders differently through nutrition or economic impacts. For example, women producers are more likely to raise poultry flocks for feeding their immediately family, which is why poultry disease outbreaks may be more likely to impact children’s nutrition (37).

### 2.4 Types of questions

Within this framework, beyond the three entry points, we categorized gender-related questions into two types: integrated and strategic. Given how little is known about how gender dynamics and norms shape AMR, we propose qualitative, exploratory questions that can help highlight the most relevant issues in each community and some of the mechanisms behind these issues (including social and gender dynamics which can be explored through the ‘how’ and ‘why’ questions included in the table). To assess the relevance of these issues the qualitative findings of these questions can be used to help shape quantitative surveys. Integrating these insights with gender-disaggregated quantitative data will be the next step to enhancing and refining this framework.

In our questions we use ‘who in the household’ as means to identify (1) which gender group is involved in a specific activity; and (2) account for other individual characteristics beyond gender that may shape involvement in the household (e.g., age, marital status, and education level). We also use ‘who in the community’ to explore gender groups intersected by other social markers that may create diverse groups in a community (e.g., ethnicity, wealth etc.) and shape their interaction with AMR (38). Finally, we refer to ‘gender groups’ to include women, men, non-binary, and intersex. We acknowledge that the experiences of the last two groups are particularly unexplored and overlooked in the context of AMR. While we recognize the need to conduct more research on these two groups, we also are aware of the complexity (and risks) associated to identifying gender groups beyond ‘women and men’ in many cultures (39). The safety of individuals must be a priority in all research which engages with any gender groups, but especially in contexts where gender groups might face stigma, discrimination or persecution (40).

## 3 Discussion: questions to improve the gender-responsiveness of AMR research in livestock systems

Using our conceptual framework (Figure 1) and gender dimension questions (Table 1), researchers working in AMR can identify gender-related questions pertinent to their programs. While these guiding questions were developed to pinpoint potential gender-related issues affecting AMR research, incorporating the unique context specificities of each research program will facilitate the generation of more tailored questions. To give an example, Table 1 question ‘Who should be able to use, access and afford antimicrobials prescribed or dispensed from animal health service (AHS)’ could be rephrased based on the local system to prescribe or dispense animal drugs. The question ‘What norms will they face?’ can be replaced by norms that are known to affect antimicrobial use in a given context. Exploration of such norms may in fact, need a separate study, given the complexity of the topic (43). This approach encourages deeper investigation and identification of relevant gender considerations.

**Table 1:** Gender dimension questions related to antimicrobial resistance research in livestock systems.

Areas impacted by gender	AMR exposure	Antimicrobial use (AMU)			AMR outcomes
Definitions	Human exposure to antimicrobial resistant pathogens during livestock production through direct contact with animals or indirect activities required for production	Access Access to antimicrobials for use in livestock production	Conservation Methods conserving antimicrobial use in livestock production including by ensuring prudent and responsible antimicrobial use, improving biosecurity measures, and using prophylactic health products (e.g. vaccines, probiotics, prebiotics)	Innovation Development and use of novel antimicrobials, antimicrobial alternatives (including vaccines) or other technologies (like diagnostics and biosecurity measures) which reduce antimicrobial use in livestock production	The productivity, economic, health, and nutritional outcomes of human infection with antimicrobial resistant pathogens during livestock production
Integrated questions	Who (in the household or community) is exposed to antimicrobial resistant pathogens in livestock production? How and why? Who is making decisions that affect AMR exposure? How and why? Who has access to information that affects AMR exposure? How and why?	Who can use, access, and afford antimicrobials in livestock production? How and why? Note: the ‘why’ here is meant to explore the social, gender and/or other dynamics that affect why some people can use, access, and afford AM, and some others cannot. Who can access and afford animal health service (AHS) providers like veterinarians or veterinary paraprofessionals in livestock production? How and why? Who is making decisions about using or purchasing antimicrobials (including from animal health service providers)? How and why? Who has access to information that affects who uses or purchases antimicrobials? How and why?	Why do livestock producers of different genders select one method of conservation over another? Why? Are there gendered risks that affect this selection? How and why? Who can use, access, and afford alternatives to antimicrobials (such as vaccines, prebiotics, and probiotics) in livestock production when such use is appropriate? How and why? Who can use, access, and afford higher quality inputs that might prevent antimicrobial use (such as high-quality feeds/supplements, improved housing) in livestock production? How and why? Who can implement, use, access and afford on-farm biosecurity measures in livestock production? How and why? Who would be able to use, access and afford animal health services or diagnostic services which ensure prudent and responsible antimicrobial use? How and why? Who is making decisions about using or purchasing antimicrobial alternatives, biosecurity measures or animal health service providers? How and why? Who has access to information that affects who uses or purchases antimicrobial alternatives, biosecurity measures or animal health service providers? How and why?	What novel antimicrobials, antimicrobial alternatives (including vaccines) or other technologies (like diagnostics and biosecurity measures) need to be prioritized for women and men livestock producers to benefit equitably? Are there gendered risks that affect how these innovations are adopted and used? How and why? What are the gendered risks and incentives associated with use of antimicrobial alternatives or implementing biosecurity measures? How and why? Who can use, access, and afford novel antimicrobials and novel alternatives to antimicrobials (such as vaccines, prebiotics, and probiotics) and other technologies in livestock production when such use is appropriate? How and why? Who would be able to use, access, and afford animal health services or diagnostic services to access novel technologies which ensure prudent and responsible antimicrobial use? How and why? Who is making decisions about using or purchasing novel antimicrobials and antimicrobial alternatives (including from animal health service providers)?? How and why? Who has access to information that affects who uses or purchases novel antimicrobials and antimicrobial alternatives? How and why?	Who is impacted by exposure or infection with antimicrobial resistant pathogens in livestock production? How (e.g. human health, economic, nutrition and livestock productivity impacts) and why? Who is making decisions that affect AMR impacts/outcomes? How and why? Who has access to information that affects AMR impacts/outcomes? How and why?
Strategic questions	What needs to be addressed to prevent gendered exposure of affected livestock producers to antimicrobial resistant pathogens?	What can be done to ensure the equitable use, access, and affordability of antimicrobials for use in livestock production when such use is appropriate? How and why? Which livestock producers wish to use antimicrobials when appropriate? What tangible constraints (such as for example, road inaccessibility; lack of transport etc.) prevent these livestock producers from using AMs? What intangible constraints (such as hostility of the community or household towards un-usual behaviour) prevent them? What needs to be addressed to allow these producers to use antimicrobials and to do so prudently? What measures/changes are needed to address both tangible and intangible constraints? Which livestock producers wish to use antimicrobials when appropriate? What needs to be addressed to allow these producers to access antimicrobials through AHSPs? What norms will they face?	What opportunities exist that can be leveraged to increase the use and adoption of methods of antimicrobial conservation among livestock producers of different genders? What needs to happen for such opportunities to benefit everyone in the household? Which livestock producers wish to use alternatives to antimicrobials when appropriate but cannot? What tangible constraints (such as for example, road inaccessibility; lack of transport etc.) prevent these livestock producers from using AM conservation measures? What intangible constraints (such as hostility of the community or household towards un-usual behaviour) prevent them? What needs to be addressed to allow these producers to use these alternatives at this time? Which livestock producers wish to use or implement biosecurity measures on farm but cannot? What are the tangible and intangible constraints affecting producers? What needs to be addressed to allow these producers to use or implement these measures at this time? What norms will they face? Which livestock producers wish to use animal health service (AHSPs) or diagnostic services which ensure prudent and responsible antimicrobial use but cannot? What needs to be addressed to allow these producers to these services? What norms will they face?	How are the prioritized innovations (development) going to impact capabilities and empowerment of livestock producers of different genders? What opportunities exist that can be leveraged to increase the use and adoption of innovations among livestock producers of different genders? Which livestock producers wish to use novel antimicrobials, alternatives to antimicrobials and technologies but cannot? What are the tangible and intangible constraints affecting their ability to use? What needs to be addressed to allow these producers to use these novel antimicrobials and alternatives at this time? Specifically, what needs to happen for both tangible and intangible constraints to be addressed. Which livestock producers wish to use novel technologies through animal health service (AHSPs) or diagnostic services which ensure prudent and responsible antimicrobial use but cannot? What needs to be addressed to allow these producers to these services? What norms will they face?	What needs to be addressed to prevent inequitable impacts of infection with resistant pathogens among livestock producers?

By targeting livestock systems, this framework addresses a critical knowledge gap. However, further efforts are required to integrate gender responsive approaches to AMR across the One Health sectors (see Galiè et al., 2024; (19)). The three entry points for gender and AMR in livestock systems discussed here are intended as starting point for further research. Additionally, the guiding questions provided are examples meant to highlight various ways gender can impact livestock systems, rather than an exhaustive list. Researchers utilizing this framework should also consider context specific questions to their production system, considering factors such as size, intensity, and species, to ensure a comprehensive understanding of gender dynamics. Similarly, age, culture, ethnicity and other intersectional factors also interact with gender; and need to be considering when applying this framework (Box 1 and Box 2).

BOX 1 **Examples of how context might impact framework applications**

Example 1: In low intensity cattle production systems in Tanzania, both men and women are actively involved in animal health management and have similar levels of disease knowledge (12). In this context, a research project focused on vaccine use might consider questions such as:

Why do cattle producers of different genders choose one vaccination over another? Are there gendered risks that influencing this selection? What opportunities exist to enhance vaccine uptake among cattle producers of different genders?

Example 2: In contrast, low intensity poultry systems in Tanzania are predominantly managed by women (41). Here, the same research project might explore questions like:

Which women poultry producers are interested in using vaccines but face barriers in doing so?

Who controls access to vaccine-related information, and what are the reasons behind these information gaps? How can access to vaccine information be improved among women poultry producers?

BOX 2 **Example of how intersectionality can impact framework applications**

Example 1: A study involving a dairy cooperative in India found that caste, as a significant intersectional factor, interacts with gender to shape power dynamics in this region (42). The research revealed that, while gender norms showed some flexibility, caste norms remained more rigid, affecting the empowerment opportunities available to women from different castes within the cooperative. A study looking at antibiotic use in these producers, key questions might include:

What intangible constraints, such as community or cooperative hostility toward unconventional practices, may limit antibiotic use among producers from different castes? What specific barriers need to be addressed to enable equitable antimicrobial access and use across caste groups?

Our next step is to implement this framework in the field. A real-life example will not only refine the framework but also reveal any limitations that may need adjustment to enhance its practical use. This will provide researchers with a concrete example of how to adapt the framework to different contexts and production systems effectively.

## 4 Conclusion

As gender-analysis in livestock research gain prominence, there is a growing expectation for AMR researchers to integrate gender considerations in their work. This framework offers a starting point for researchers aiming to enhance gender inclusivity and considerations in AMR research and policy development within livestock systems. To advance gender mainstreaming in future AMR research projects and policy implementation in livestock and other systems, researchers need to embrace gender-responsive research methodologies.

## Data Availability

The original contributions presented in the study are included in the article/supplementary material, further inquiries can be directed to the corresponding author.
